# Changes in sub-cellular localisation of trophoblast and inner cell mass specific transcription factors during bovine preimplantation development

**DOI:** 10.1186/1471-213X-13-32

**Published:** 2013-08-13

**Authors:** Zofia E Madeja, Jaroslaw Sosnowski, Kamila Hryniewicz, Ewelina Warzych, Piotr Pawlak, Natalia Rozwadowska, Berenika Plusa, Dorota Lechniak

**Affiliations:** 1 Department of Genetics and Animal Breeding, Poznan University of Life Sciences, Wolynska 33, Poznan 60-673, Poland; 2 Department of Reproduction and Stem Cells, Institute of Human Genetics, Polish Academy of Sciences, Poznan, Poland; 3 Faculty of Life Sciences, The University of Manchester, Manchester, UK

**Keywords:** Bovine blastocyst, ICM/TE lineage segregation, Cell fate, Gene expression patterns, CDX2, Mitotic retention

## Abstract

**Background:**

Preimplantation bovine development is emerging as an attractive experimental model, yet little is known about the mechanisms underlying trophoblast (TE)/inner cell mass (ICM) segregation in cattle. To gain an insight into these processes we have studied protein and mRNA distribution during the crucial stages of bovine development. Protein distribution of lineage specific markers OCT4, NANOG, CDX2 were analysed in 5-cell, 8–16 cell, morula and blastocyst stage embryos. ICM/TE mRNA levels were compared in hatched blastocysts and included: *OCT4*, *NANOG*, *FN-1*, *KLF4*, *c*-*MYC*, *REX1*, *CDX2*, *KRT*-*18* and *GATA6*.

**Results:**

At the mRNA level the observed distribution patterns agree with the mouse model. CDX2 and OCT4 proteins were first detected in 5-cell stage embryos. NANOG appeared at the morula stage and was located in the cytoplasm forming characteristic rings around the nuclei. Changes in sub-cellular localisation of OCT4, NANOG and CDX2 were noted from the 8–16 cell onwards. CDX2 initially co-localised with OCT4, but at the blastocyst stage a clear lineage segregation could be observed. Interestingly, we have observed in a small proportion of embryos (2%) that CDX2 immunolabelling overlapped with mitotic chromosomes.

**Conclusions:**

Cell fate specification in cattle become evident earlier than presently anticipated – around the time of bovine embryonic genome activation. There is an intriguing possibility that for proper lineage determination certain transcription factors (such as CDX2) may need to occupy specific regions of chromatin prior to its activation in the interphase nucleus. Our observation suggests a possible role of CDX2 in the process of epigenetic regulation of embryonic cell fate.

## Background

Despite recent advances in developmental biology our understanding of cell fate decisions during preimplantation development of non-rodent mammals still remains limited. With growing prospects of the use of stem cell research in medicine and animal biotechnology, cell lineage formation in species of economic significance, such as cattle or sheep is of increasing interest. Sharing many similarities with human embryos, bovine embryos present an attractive model for investigating the fundamental mechanisms of early development. These include the timing of epigenetic reprogramming, the stage of the embryonic genome activation and the timing of development [1,2].

During the initial stages of mammalian development two successive differentiation events lead to the segregation of the three committed cell lineages. The first event begins at the compacted morula stage, where the outer layer of cells segregates to become the epithelial trophectoderm (TE) that gives rise to the embryonic part of the placenta. The inner layer of cells (inner cell mass, ICM) will produce the embryonic lineages. The second round of segregation divides the ICM into primitive ectoderm, which gives rise to the epiblast (nascent embryo proper) and the primitive endoderm (PrE) that forms the extra-embryonic endoderm layer of the visceral yolk sac and in rodents also the parietal endoderm. The first cell fate decisions are governed by a combination of interactions between transcriptional networks, signalling, cell polarity and cell position within the morula [3]. At the cellular level the ICM/TE segregation is initiated by two rounds of differentiative divisions that result in spatially and molecularly distinct cell populations. The differentiation of TE may be regarded as the hallmark event in mammalian preimplantation development, as it is the first tissue that becomes differentiated during embryogenesis. Due to its biological function the ICM needs to retain its pluripotency status much longer, since the establishment of the three germ layers (endoderm, mesoderm, ectoderm) occurs soon after implantation in rodents and around day 14 in cattle [4]. In mice the process of TE formation initiates at the 8-cell stage, when embryos undergo compaction manifested by an increased intracellular adhesion, blastomere flattening and formation of adherens junctions [5]. Concurrent with this event, cells acquire an apical domain rich in proteins, such as the atypical protein kinase aPKC [6], the polarity protein Par3 [7] and the apical protein Erizn [8]. During the subsequent cell divisions (from 8–16 and from 16–32 cell stage) the differential inheritance of the apical, polarised surface determines either the TE or the ICM fate [9,10].

At the molecular level the initiation of the TE lineage formation is promoted by the action of Tead4, which was shown to regulate multiple transcription factors important for trophoblast development [11,12]. Trophoblast maintenance depends on consecutive activation of factors such as caudal-type homeodomain transcription factor 2 (Cdx2), trans-acting GATA binding transcription factor (Gata3), eomesodermin (Eomes) and epithelial-specific ETS factor (Elf5) ([13-18], rev. [19]). Cdx2 is the earliest known marker used to distinguish between mouse TE and ICM. Its expression begins at the 8-cell stage and becomes restricted to the outside cells at the time of blastocyst formation [15,20]. The polarity of the outer blastomeres is possibly the main factor responsible for up-regulation of Cdx2 expression in these cells [21]. Once Cdx2 expression becomes restricted to the outer cells, factors related to the maintenance of pluripotency and the ICM lineage formation are repressed. This action is maintained through a feedback loop regulated by Elf5 via the mutual suppression between Cdx2 and octamer binding transcription factor 3/4 (Oct3/4) and homeobox transcription factor (Nanog) complexes. Functional studies showed that Cdx2 blocks Oct3/4 activity in the TE committed cells, as in the presence of Cdx2, Oct3/4 occupied the heterochromatic regions of the nuclei [22]. More importantly, it was shown that Cdx2 can post-transcriptionally inactivate Oct4, thus TE commitment may occur before Oct4 transcription is terminated [22,23]. The establishment of the ICM and the epiblast relies on the interactions of transcription factors Oct4, Nanog, Sall4 and Sox2 ([24-27], rev. [28]). These factors are also key players in maintaining embryonic stem cell (ESC) pluripotency and possess an ability to auto-regulate their own transcription by sustaining a self-reinforcing transcriptional network. Sox2 can synergistically act with Oct3/4 to activate Oct-Sox enhancers, which in turn regulate the expression of pluripotent stem cell specific genes such as Nanog, Oct3/4 and Sox2 [29]. Inactivation of Nanog results in peri-implantation lethality as mouse blastocysts fail to form the epiblast. Moreover, Nanog-null embryos exhibit a PrE marker Gata6 throughout the ICM [25,30] and knockdown of *Nanog* gene in mouse blastocysts and ESC resulted in an up-regulation of the TE specific genes (Cdx2 and Hand1) [31].

The unique molecular interactions underlying the ICM/TE lineage specification in cow and other ungulate species still remains to be described in detail. The work by Berg et al. [32] shows that OCT4 expression in cattle TE may be retained until the PrE/epiblast segregation, indicating that the trophoblast cells may not become committed during bovine pre-implantation development at stages developmentally equivalent to the mouse. It was shown that at the early stages, CDX2 does not repress OCT4 expression, as the bovine OCT4 locus does not contain the *cis*-acting regulatory region necessary for extinguishing its transcription in TE [32]. Moreover, it was indicated that the TE cells of an early bovine blastocyst (7dpi) retain the ability to contribute to the ICM derivatives in chimeras [32]. It is therefore plausible that the standard ICM/TE markers described in the mouse become segregated at different (or later) stages during the ruminant embryo development.

Using cattle as a non-rodent model animal we have aimed to describe the mutual spatial allocation of factors that have been shown to be important for lineage commitment in the mouse. We have followed the specific distribution patterns from the very early stages of development, through the time of embryonic genome activation (EGA), until the hatched blastocyst (HBl) stage. Contrary to what is claimed by the majority of published reports, we suggest that cell fate decisions in cattle may become initiated earlier than it is presently anticipated – around the time of embryonic genome activation. Of most interest our study indicates that CDX2 could possibly be subject to the phenomenon of mitotic retention, which may imply a role in epigenetic regulation of embryonic cell fate.

## Methods

### *In vitro* production of bovine embryos (IVP)

All procedures were performed in accordance with the guidelines of the National Ethical Commission for Animal Research (Ministry of Science and Higher Education, Poland). The study was approved by the Local Ethical Commission (dr Z.E. Madeja personal licence permit number: 142/2010).

Unless stated otherwise, all reagents used for IVP culture media preparation were supplied by Sigma-Aldrich, Poland. The media were made based on sterile embryo-tested water (Gibco, Life Technologies Poland).

Bovine cumulus-oocyte-complexes (COC) were collected from slaughterhouse ovaries. COCs were aspirated from 2–6 mm follicles, underwent a restrictive selection process [33] and were subjected to *in vitro* maturation (IVM). COCs were matured for 24 hours in TCM-199 medium containing 1 mg/ml fatty acid free BSA (fafBSA), 0.05 mg/ml gentamycin, 0.022 mg/ml Na-pyruvate, 2.2 mg/ml NaHCO_3_ and hormones (5 UI/ml hCG, 10 UI/ml PMSG, Intervet) at 39°C in humidified atmosphere with 5% CO_2_ (as described by Stinshoff et al. [34]). Insemination was done with bull sperm supplied by the Centre for Animal Breeding and Reproduction (Poznan, branch Tulce; 63–004 Tulce, Poland) at a concentration of 1 × 10^6^/ml. The sperm were washed twice by centrifugation and re-suspended in standard IVF-Sperm-Talp medium supplemented with 4 mg/ml fafBSA [35]. PHE (penicillin, hypotaurine, epinephrine) was used for sperm capacitation. After 20 hours of gamete co-incubation at 39°C in humidified atmosphere with 5% CO_2_, the cumulus cells were mechanically removed by pipetting. The presumptive zygotes were transferred in groups of 10 to 30 μl culture drops covered by embryo culture tested mineral oil. *In vitro* embryo culture (IVC) was carried out at 39°C in humidified atmosphere of 5% CO_2_, 5% O_2_, 90% N_2_ in a modular chamber placed inside an incubator. The IVC medium consisted of synthetic oviduct fluid culture medium (SOF) supplemented with essential and non-essential amino acids (MEM, BME) and 4 mg/ml fafBSA, as described by Holm et al. [36]. At 3dpi the embryos were subjected to selection. Only cleaved embryos were placed in fresh IVC medium and were left in culture until they had reached the developmental stage required for the experiments.

### Embryo collection for qualitative and quantitative PCR analysis

The selected material was washed with 0.25% polyvinylpirrolidone (PVP) in PBS placed in 1.5 ml tubes in minimal volume, frozen in liquid nitrogen and stored in −80°C.

*Qualitative gene expression analyses* were performed on bovine embryos at 8–16 cell stage, morula stage and hatched blastocysts (9dpi). 8–16 cell embryo samples were collected in pools, each consisting of 20 embryos. Morulae were collected in groups of 12 and 3 un-dissected blastocysts were pooled to make up one sample. We have collected 6 independent samples for each stage, collected over several IVP experiments. This allowed to eliminate the possible bias in gene expression analyses which could distort the results if all samples were collected during one IVP experiment.

*Quantitative gene expression analysis* were performed on hatched blastocysts 9dpi. In order to reveal the possible differences in gene expression levels between the ICM and the TE, the blastocysts were microsurgically dissected. The ICMs made up one sample and the corresponding TEs made up the other sample. The ICM/TE samples were pooled in groups of 3 as we have empirically established that it was the lowest material content that allowed for efficient RNA extraction, cDNA synthesis and reliable real-time PCR analysis. In total we have collected 24 independent samples (12 ICM and 12 TE) from 72 embryos. For the means of quantitative calculations we have also collected 6 samples each containing 3 whole (un-dissected) HBls, that served as calibrators for the purpose of transcript quantification.

### RNA extraction and cDNA synthesis

Total RNA was extracted from embryos using the High Pure miRNA Isolation Kit (Roche Diagnostics, Poland) according to the manufacturer’s protocol. This kit was developed for the extraction of small RNA molecules, and it has proven to be an effective tool in extracting RNA from small copy number samples such as the preimplantation embryos. RNA concentration was measured on NanoDrop (Thermo Scientific, USA). For each sample reverse transcription was performed from 100 ng of total RNA. cDNA synthesis was carried out with the Transcriptor High Fidelity cDNA Synthesis Kit (Roche Diagnostics) according to the manufacturer’s protocol. The samples were stored in −20°C.

### Quantitative real-time RT-PCR analysis

In short, we have compared the expression level of a certain gene in ICM and TE and related it to an un-dissected embryo. The experiments were performed on Roche Light Cycler 2.0 instrument. The calculations were based on relative gene expression data analyses using real-time PCR and the 2^-ΔΔC^_T_ method as described by Livak and Schmittgen [37] and Schmittgen and Livak [38]. In order to establish the differences in gene expression levels between the ICM and the TE, the samples containing whole blastocysts served as reference points for relative gene expression level calculations. HBL was referred to as a calibrator. Gene expression levels were normalised to the expression level of the 18S rRNA gene that served as a reference. Each sample was subjected to gene expression analyses with all of the primer sets designed for the panel of chosen lineage specific genes and for the reference gene. All reactions were repeated in triplicates. The primers were designed to span the two neighbouring exons (Table 1). The reactions were carried out in 10 μl capillaries (Roche Diagnostics) and the PCR mix comprised of 1 μl of Light Cycler Fast Start DNA master SYBR Green (Roche Diagnostics), 5 mM MgCl_2_ (Roche Diagnostics), 0.3 μM of primers and 1 μl of cDNA. Real-time PCR reaction conditions included: initial polymerase activation at 95°C for 10 min, followed by 42 cycles of denaturation at 95°C for 10 s, annealing (primer specific, listed in Table 1) for 10 s, elongation at 72°C for 20 s. Product specificity was confirmed by melting analysis.

**Table 1:** **Primer pairs and annealing conditions used for the real**-**time PCR gene expression analyses**

**Name**	**Sense primer**	**Antisense primer**	**Product size**	**Annealing temperature**
*CDX2*	CTTTCCTCCGGATGGTGATA	AGCCAAGTGAAAACCAGGAC	113 bp	58°C
*c-MYC*	TGGACGCTAGATTTCCTTCG	GCTGCTGCTGGTGGTAGAAG	155 bp	58°C
*FN1*	GCACTATGGCCAAAGAGAGG	AACAGGAGACAAGGGTGTGG	109 bp	58°C
*KLF4*	AAACCAAAGAGGGGAAGACG	ATGTGTAAGGCGAGGTGGTC	291 bp	58°C
*KRT18*	GAGATCGAGGCTCTCAAGGA	GTCCAGCTCCTCTCGGTTCT	201 bp	58°C
*NANOG*	AAACAACTGGCCGAGGAATA	AGGAGTGGTTGCTCCAAGAC	194 bp	58°C
*OCT4*	GTTTTGAGGCTTTGCAGCTC	CTCCAGGTTGCCTCTCACTC	185 bp	58°C
*GATA6*	CTGCGGTCTCTACAGCAAGA	GTGGTCGTGGTGTGACAGTT	117 bp	58°C
*REX1*	GGAAGAGGACCCACTCCTTC	ACTTGGCCTCCTAGTGCATC	241 bp	58°C
*18S rRNA*	GGAGAGGGAGCCTGAGAAAC	TCGCGGAA GGATTTAAAGTG	170 bp	58°C

### Statistical analysis

A certain level of heterogeneity was noted within the analysed sample groups, thus the data did not follow normal distribution. Therefore to analyse statistically significant variability in gene expression levels between ICM and TE, the Wilcoxon signed-rank test was applied. Due to the nature of the experiment (number of repeats of each individual sample within one group – ICM/TE) the data was processed as median and presented as ± SEM. Differences of P < 0.05 were considered significant. The mRNA data expression was analysed using the statistical package SPSS 17.0.

### Whole mount immunofluorescence

The material included embryos at stages crucial for bovine development: 5-cell, 8-16-cell (bovine embryonic genome activation), morula (compaction) and blastocysts at various developmental stages - early blastocyst (7dpi), expanding blastocyst (8dpi) and hatched blastocyst (9dpi) as classified by Rekik et al. [39]. The embryos were selected during the same IVP experiments as the material collected for qualitative and quantitative gene expression analyses (Table 2).

**Table 2:** **Experimental groups and antibody set**-**up for single and double immunolabelling**

**Developmental**	**5-cell (n)**	**8-16 cell (n)**	**Morula (n)**	**Blastocyst**	**Blastocyst**	**Hatched**
**Stage**				**7dpi (n)**	**8/9dpi (n)**	**Blastocyst**
						**9dpi (n)**
**Number of immunostained embryos per staining set-up (n)**				1 (5)		1 (5)
			1 (4)	2 (4)		2 (5)
	1 (10)	1 (5)	2 (4)	3 (4)		3 (6)
	2 (10)	2 (4)	3 (4)	4 (4)		4 (5)
	3 (10)	3 (4)	4 (4)	5 (4)	6 (5)	5 (6)
	4 (10)	4 (4)	6 (6)	6 (9)	7 (5)	6 (12)
	5 (10)	5 (5)	8 (4)	7 (8)	10 (10)	7 (11)
		9 (20)	9 (11)	8 (6)		8 (13)
			10 (5)	9 (4)		9 (10)
				10 (8)		10 (12)
**Antibody staining set-up**	1 – CDX 2 (ab15258) mouse			6 – CDX 2 (ab15258) mouse + OCT4 (ab18976) rabbit		
	2 – CDX 2 (ab88129) rabbit			7 – CDX 2 (ab88129) rabbit + OCT4 (ab27985) goat		
	3 – OCT4 (ab27985) goat			8 – CDX 2 (ab15258) mouse + NANOG (500-P236) rabbit		
	4 – OCT4 (ab18976) rabbit			9 – CDX 2 (ab15258) mouse + H3K9me3 (07–442) rabbit		
	5 – NANOG (500-P236) rabbit			10 – OCT4 (ab27985) goat + NANOG (500-P236) rabbit		

From embryos of developmental stages earlier than HBL, zona pellucida (ZP) was removed prior to fixation by a brief incubation in acid Tyrode’s solution supplemented with 0.1 N HCl. The embryos were processed as follows: briefly washed in PBS (pH 7.3) and fixed with 4% paraformaldehyde in PBS with 0.1% Triton X-100 and 0.1% Tween20 (pH 8.0) for 15 min at 39°C (Sigma Aldrich). Immunofluorescent protein labeling was carried out as previously described by Madeja et al. [40] and Plusa et al. [41] with some modifications: permeabilisation with 0.55% Triton X-100/PBS for 20 min; blocking of non-specific antibody binding with 10% fetal calf serum in PBS, 60 min at room temperature (RT); three washes in 0.1% Triton X-100/PBS (PTX); overnight incubation with primary antibodies (ABI) diluted 1:50 in blocking buffer at 4°C; three washes in PTX; blocking for 40 min at RT; secondary antibody (ABII) incubation at 1:200 dilution; three washes in PTX; embryo mounting on concave glass slides in 40 μl drop containing antifade solution with DAPI (Vectashield mounting medium, Vector Laboratories, USA). The slides were stored at +4°C.

To avoid cross-reactivity between the AB hosts the embryos were double labeled with various sets of ABI and ABII. **The primary antibody set included: anti-CDX2** - mouse monoclonal AB (Abcam, UK, ab15258) or rabbit polyclonal AB (Abcam, ab88129); **anti-OCT4** rabbit polyclonal AB (Abcam, ab18976) or goat polyclonal AB (Abcam, ab27985); **anti-NANOG** rabbit polyclonal AB (PeproTech, UK, 500-P236), **anti-trimethyl-histone H3 (Lys9)** AB (Merck-Millipore, 07–442). **The secondary antibody set consisted of** Santa Cruz Biotechnology, USA, **ABII:** donkey anti-goat Rhodamine conjugated AB (sc-2094) or donkey anti-goat FITC conjugated AB (sc-2024), donkey anti-mouse Rhodamine conjugated AB (sc-2300); donkey anti-rabbit FITC conjugated (sc-2090), chicken anti-rabbit Rhodamine conjugated AB (sc-2862), chicken anti-mouse FITC conjugated AB (sc-2989); goat anti-mouse Rhodamine conjugated AB (sc-2092), goat anti-mouse FITC conjugated AB (sc-2010), goat anti-rabbit Rhodamine conjugated AB (sc-2091), goat anti-rabbit, FITC conjugated (sc-2012). **Blocking peptide** for CDX2: Abcam (ab99158). Fluorescent signals were visualized using a Zeiss Axiovert 200 M laser scanning confocal microscope.

## Results

### Gene expression patterns of key lineage specific marker genes during bovine preimplantation development

**Figure 1:**
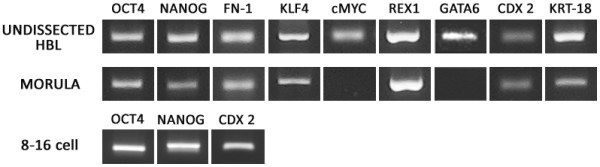
**Qualitative gene expression profiles of lineage markers in bovine 8**–**16**, **morula and HBl stage embryos.**

In order to follow lineage specification during bovine preimplantation development we have first analysed the mRNA expression profile of selected pluripotency and lineage specific markers: *OCT4*, *NANOG*, *FN*-*1*, *KLF4*, *c*-*MYC*, *REX1*, which are important for maintaining pluripotency in mouse and human and *CDX2*, *KRT*-*18* which are crucial for the establishment and the maintenance of TE as well as *GATA6* involved in mouse PrE lineage segregation. Initially we have performed qualitative gene expression analyses to establish which genes were present in cattle morula and blastocyst stage embryos. Transcripts for all of the mentioned genes were detected in blastocysts and all except *c*-*MYC* and *GATA6* were present in the morula stage embryos (Figure 1). Because for protein immunolocalisation we have chosen to concentrate on the 3 canonical lineage markers OCT4, NANOG and CDX2, we have also checked the presence of gene specific mRNAs in 8–16 cell stage embryos, which in bovine is the time of major embryonic genome activation (Figure 1).

#### The expression patterns of key lineage specific marker genes in bovine blastocysts 9dpi resembles the classical mouse model

In order to establish whether the investigated transcription factors were differentially expressed between bovine ICM and TE we have concentrated on the latest developmental stage that may be obtained *in vitro* under standard culture conditions – the hatched blastocyst stage (HBl, 9dpi). To separate the ICM from the TE, the embryos were microsurgically dissected. This method allowed collection of comparable samples as, unlike in rodents and higher primates, the bovine ICM is a clearly pronounced structure where polar TE quickly disappears after blastocyst expansion [42].

**Figure 2:**
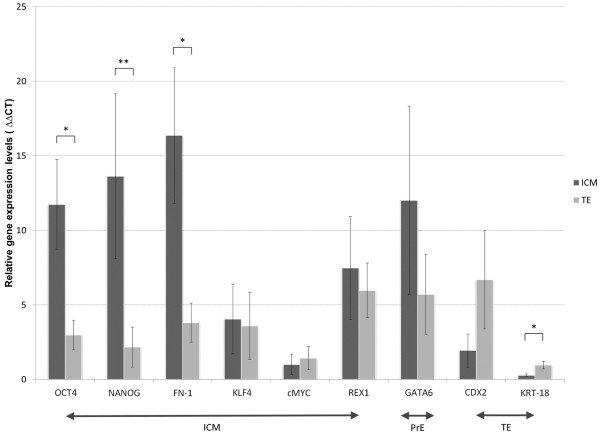
**Comparison of gene expression levels between bovine ICM and TE.** The graph represents relative expression levels of pluripotency and lineage specific genes in bovine ICM and TE. Asterisks denote significant differences in mRNA levels: * P < 0.05; ** P < 0.01. Error bars represent SEM.

The results revealed significantly higher expression levels of *OCT4*, *NANOG* and *FN*-*1* in bovine ICM as compared to TE (Figure 2). *OCT4* mRNA level showed a 4-fold up-regulation in the ICM (P = 0.05) and *NANOG* and *FN*-*1* gene expression levels were 6-fold (P = 0.002) and 4-fold (P = 0.015) higher in the ICM respectively. Out of 12 TE samples 6 did not express *NANOG*. The persistent expression of *NANOG* was possibly related to slight differences in the developmental timing of some of the blastocysts, as bovine blastocyst hatching is variable and takes place between 7.5dpi and 11dpi [43,44]. In parallel, trophoblast related factors *CDX2* and *KRT*-*18* also displayed lineage specific mRNA distribution pattern. In 9 out of 12 ICM samples *CDX2* transcripts were not detected. Overall *CDX2* expression level was 3-fold higher in bovine trophoblast versus the ICM. However the difference was not statistically significant (P = 0.071). The expression levels of *KLF4*, *c*-*MYC* and *REX1* showed no specific differences between the ICM and the TE. *GATA6* expression profile pointed towards its higher abundance in the ICM (2-fold), however a notable level of variability between the samples was observed (SEM = 6.33).

### ICM specific transcription factors exhibit dynamic changes in subcellular localisation during bovine development

**Figure 3:**
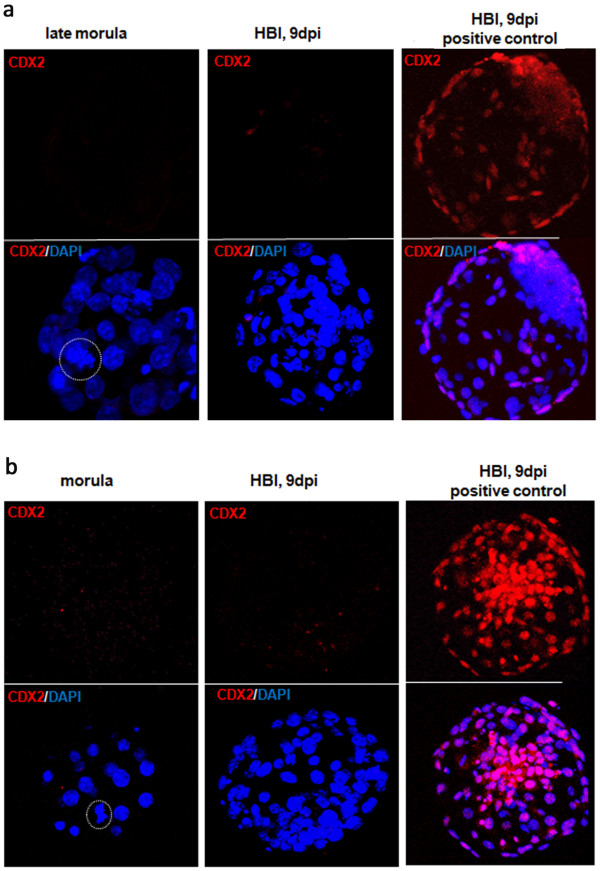
**CDX2 immunolabelling controls.** The binding specificity of the rabbit polyclonal anti CDX2 primary antibody (ab88129) **(a)** and mouse monoclonal anti CDX2 primary antibody (ab15258) **(b)** was verified with the use of blocking peptide (ab99158) prior to the antibody staining. The secondary antibodies used were goat anti-rabbit Rhodamine conjugated (sc-2091) in **(a)** and goat anti-mouse Rhodamine conjugated (sc-2092) in **(b)**. Dashed circle marks the metaphase plate. DAPI labels chromatin.

**Figure 4:**
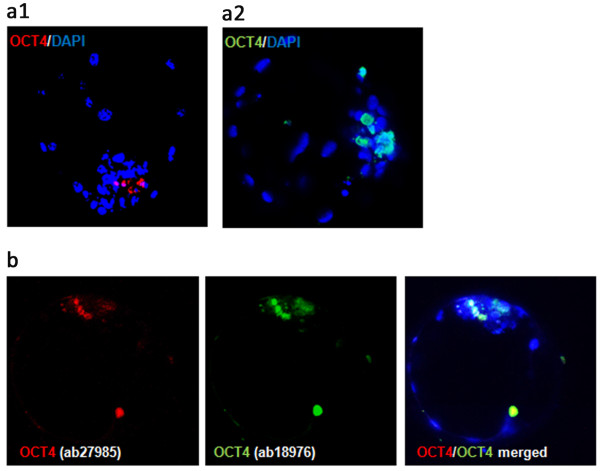
**OCT4 immunolabelling controls.** Control immunostaining with both of the anti OCT4 antibodies used in the experiment. **(a1)** presents HBL single labelled for OCT4 with goat polyclonal primary antibody (ab27985) detected with mouse anti-goat Rhodamine conjugated secondary antibody (sc-24900). **(a2)** presents single labelled HBl with rabbit polyclonal anti OCT4 antibody (ab18976) detected with goat anti-rabbit FITC conjugated secondary antibody (sc-2012). **(b)** indicates colocalisation of the OCT4 in HBl double-labelled with both of the anti OCT4 antibodies. The secondary antibodies used were mouse anti-goat Rhodamine conjugated (sc-2490) and donkey anti-rabbit FITC conjugated (sc-2090). DAPI labels chromatin.

To complement the observations made from studies of ICM and TE transcription profiles we have immunolabelled the classical lineage and pluripotency marker proteins during bovine preimplantation period from the 5-cell stage until the hatched blastocyst stage (9dpi). Embryos were double labelled for various combinations of proteins: OCT4, NANOG and CDX2, which allowed examination of their mutual spatial correlation (Table 2). The control groups underwent the same staining protocol as the experimental group, with the exception of the primary antibody (the results are provided in the Additional file 1: Figure S1 section). The specificity of the CDX2 primary AB binding was verified with the use of blocking peptide (Figure 3). OCT4 binding was validated by performing double immunolabelling with both of the anti OCT4 antibodies used (Figure 4). The application of anti NANOG (500-P236) and anti OCT4 (ab27985) antibody has been proven by Puy et al. [45] and He et al. [46].

#### Inner cell mass lineage allocation

**Figure 5:**
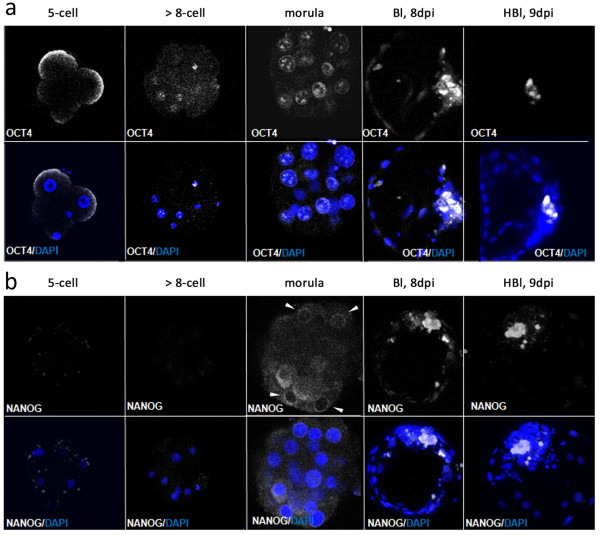
**Distribution of OCT4 and NANOG in the early stages of bovine development.** Immunofluorescent detection of OCT4 **(a)** and NANOG **(b)** followed through the early stages of bovine development. In 5-cell embryos OCT4 **(a)** localised at the apical surface of the blastomeres, and from the 8–16 cell stage weak signal was detected in cell nuclei and mostly absent in the cytoplasm. NANOG **(b)** immunostaining was for the first time detected at the morula stage exhibiting a specific cytoplasmic localisation with characteristic rings around the nuclear periphery (indicated by arrow heads). From the early stages of blastocyst formation OCT4 and NANOG signals were detected in the nuclei of the ICM cells. Confocal sections were taken every 3 μm. Chromatin was labelled with DAPI.

**Figure 6:**
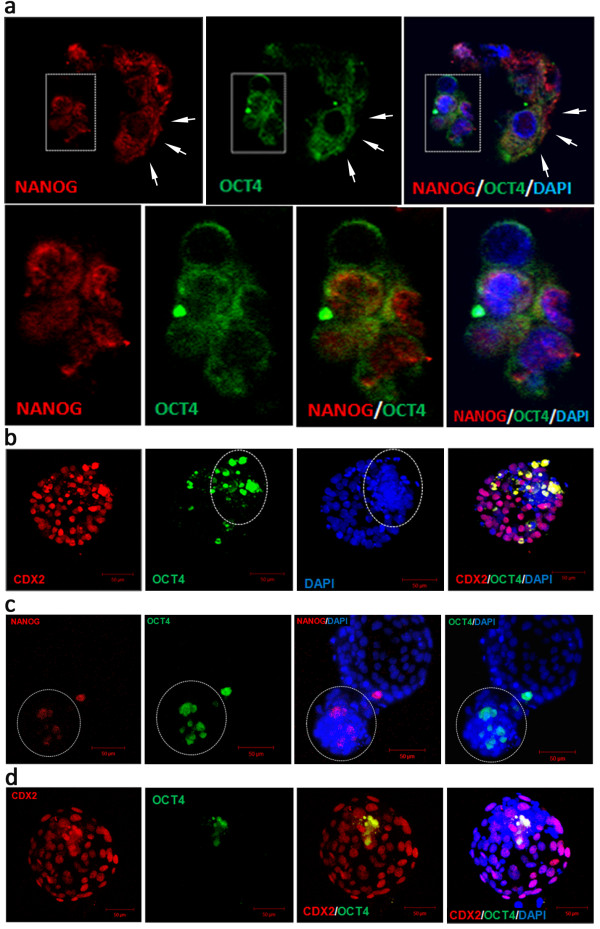
**ICM lineage segregation during bovine blastocyst formation. (a)** presents a single optical section through an ICM of bovine early blastocyst (7dpi) immunostained for NANOG and OCT4. The boxed areas are enlarged in the bottom row. NANOG shows nuclear and OCT4 sub-nuclear localisation in ICM White arrows indicate cytoplasmic localisation of NANOG and OCT4 in the trophoblast cells in the vicinity of the ICM. Confocal sections were taken every 2 μm **(b)** shows a 3-D reconstruction of confocal images of an early blastocyst (7/8dpi) double labelled for CDX2 and OCT4. OCT4 was predominantly located in the ICM, but some signal was detected in the TE cells. Dashed circle marks the ICM. **(c)** is a 3-D compilation of optical sections taken through hatching blastocyst (8/9dpi) labelled for NANOG and OCT4. Dashed circle marks the ICM. **(d)** shows a 3-D reconstruction composed of 30 confocal sections (taken every 3 μm) through bovine hatched blastocyst (9dpi) double labelled for CDX2 and OCT4. At this stage OCT4 positive cells entirely segregate to the ICM. CDX2 becomes restricted to the trophoblast. DAPI marks chromatin. Confocal sections were taken every 3 μm.

**Figure 7:**
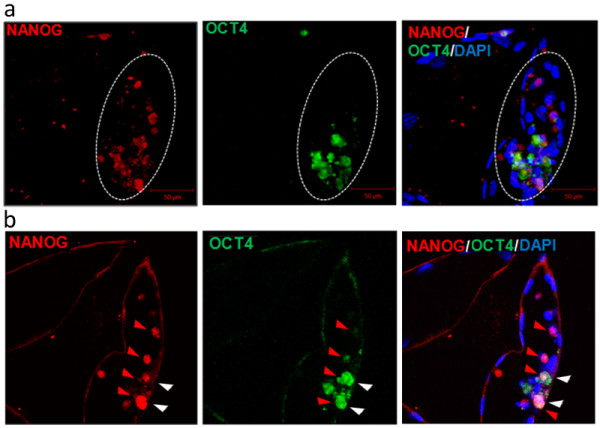
**OCT4 and NANOG distribution pattern in bovine hatched blastocysts (9dpi).** The top panel **(a)** of images shows a 3-D reconstruction of confocal sections taken through bovine HBL. Dashed line encircles the ICM. The bottom panel **(b)** represents a single optical section through the ICM of the same embryo. OCT4 and NANOG localise in the ICM, but a certain degree of signal segregation was observed. Cells indicated with red arrowheads express NANOG and cells indicated with white arrowheads co-express both proteins. DAPI marks chromatin. Confocal sections were taken every 3 μm.

OCT4 and NANOG protein distribution patterns were investigated during the crucial stages of bovine preimplantation development. NANOG was not detected in 5-cell and 8–16 cell stage embryos, however as revealed by qualitative gene expression analysis, transcripts for both of these proteins were present (Figure 1). OCT4, the key transcription factor necessary to maintain the ICM, at the 5-cell stage occupied the apical surface of the blastomeres (a pattern identified in all of the analysed embryos). Around the 8-cell stage, the protein could be for the first time in development, located in the nuclei of all cells (Figure 5a). The situation changed at the morula stage, where both OCT4 and NANOG could be detected, however the distribution patterns were different. NANOG was localised in the cytoplasm of some cells, forming characteristic rings around the nucleus (Figure 5b, arrowheads). This data, combined with mRNA detection, suggests that NANOG protein appeared *de novo* at the morula stage as a product of the embryonic genome. Moreover NANOG positive cells seemed to be located in one area of the embryo (possibly the nascent ICM). At the same developmental stage OCT4 produced a nuclear signal in all cells. In early blastocysts (7dpi) OCT4 and NANOG proteins were found to be located both in the ICM and in the surrounding TE cells, however their distribution patterns were different. In the ICM cells both NANOG and OCT4 displayed a nuclear localisation, however NANOG occupied the whole territory of the nucleus, whereas OCT4 showed more peripheral localisation, with “rings” at the nuclear periphery of the ICM cells (Figure 6a). In trophoblast cells, where OCT4/NANOG activity is redundant, proteins remained in the cytoplasm (Figure 6a, arrows). At 7/8dpi OCT4 positive blastomeres predominantly located in the ICM, however some positive signal was still detected within the TE cell population (Figure 6b). At the time of blastocyst hatching, still some residual immunolabelling could be observed in the TE layer, as indicated by a single cell co-expressing NANOG and OCT4 in Figure 6c. Following OCT4/CDX2 distribution patterns at the stages of blastocyst formation we have concluded that the process of ICM specific OCT4 allocation is gradual. Concomitant with blastocyst expansion at 8dpi OCT4 expressing cells become limited to the ICM, however single positive cells could be still identified in the TE of about 50% of the analysed embryos. After blastocyst hatching we have noted further evidence for lineage segregation. Both OCT4 and NANOG became exclusively ICM specific (Figure 6d). Within the group of cells expressing OCT4 and NANOG, we have identified cells expressing only NANOG (as indicated by red arrowheads in Figure 7b) and cells expressing both factors, with NANOG signal being predominantly nuclear and OCT4 showing some nuclear, some cytoplasmic localisation (white arrowheads in Figure 7b).

#### Trophoblast specific factor CDX2 changes its sub-nuclear localisation during bovine preimplantation development

**Figure 8:**
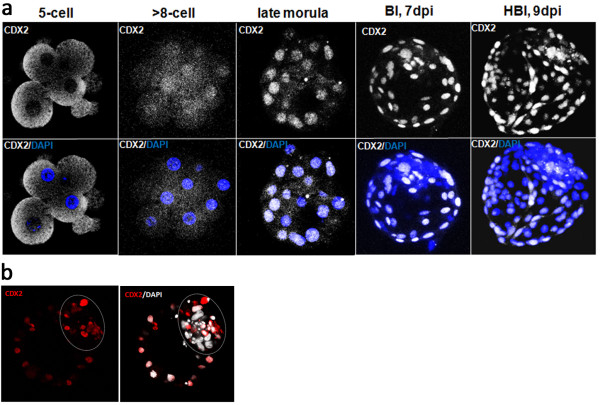
**Distribution of CDX2 during the early stages of bovine development. (a)** presents the specific changes in the CDX2 protein distribution from the 5-cell stage. Initially CDX2 was located in the cytoplasm of all cells and was absent in the nuclei. From 8-cell onwards CDX2 specifically allocated to the embryonic cell nuclei and became TE specific at the blastocyst stage. **(b)** shows a single optical section through the early stage blastocyst (7/8dpi) already presented in Figure 5a. At this stage CDX2 immunostaining was detected in the TE with some residual staining still visible in the ICM (highlighted by the dashed circle). DAPI marks chromatin. Confocal sections were taken every 3 μm.

To follow the process of bovine TE lineage specification, we have examined CDX2 distribution profile in 5-cell, 8–16 cell, morula and blastocyst stage embryos (7-9dpi). In 5-cell embryos CDX2 was located in the cytoplasm of all cells (Figure 8a) and unlike OCT4 showing a more pronounced distribution within the blastomeres and absence from the nucleus. At the subsequent stages of development CDX2 specifically allocated to the embryonic cell nuclei (Figure 8a). TE lineage specification becomes evident at more advanced developmental stages. At 7/8 dpi CDX2 segregation to the trophoblast cells could already be noted, however some signal was still detected within the ICM cells (Figure 8b) as indicated by a confocal section through an early blastocyst presented in Figure 6b.

**Figure 9:**
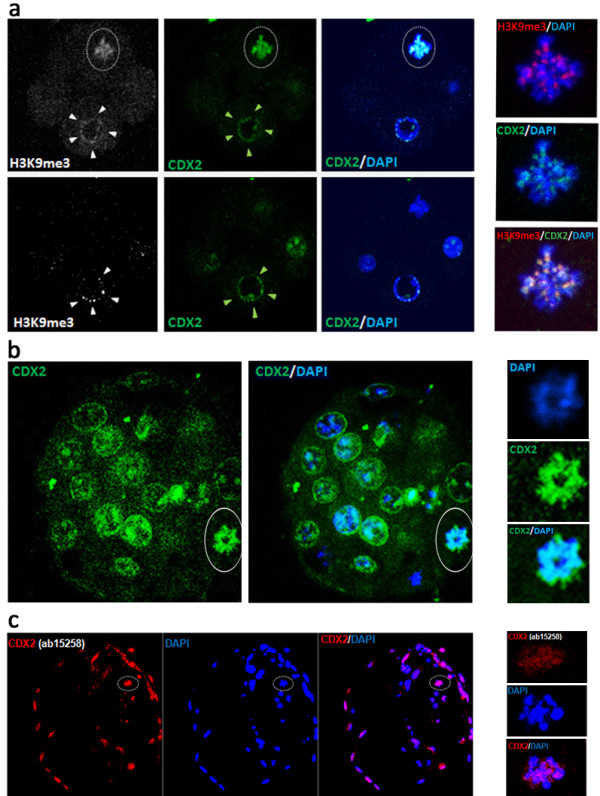
**CDX2 displays nuclear localisation and labels metaphase chromosomes. (a)** presents 2 consecutive optical sections through an early bovine embryo double labelled for CDX2 and for histone lysine methylation H3K9me3. White arrow heads point to a typical heterochromatin distribution pattern at the periphery of the interphase nucleus. Green arrow heads indicate CDX2 and H3K9me3 colocalisation. Dashed circle in **(a)** highlights the metaphase plate. CDX2 signal overlays the chromosomes and colocalises with condensed, transcriptionally silent chromatin as indicated by H3K9me3 immunostaining enlarged in the right panel. **(b)** presents an optical scan through middle of late morula embryo (beginnings of cavity formation may be visible in top section of the embryo). CDX2 displays nuclear localisation and also labels the metaphase chromosomes (marked by the dashed white circle, the area is enlarged in the boxed panel). **(c)** HBl (9dpi) immunostained for CDX2. Dashed white circle highlights the metaphase plate where, the encircled area is enlarged in the boxed panel where the CDX2 signal colocalisation with metaphase chromosomes is visible. DAPI marks chromatin. Confocal sections were taken every 3 μm.

A detailed analysis of the confocal images of the morula stage embryos indicated that the distribution pattern within the nucleus was not uniform. CDX2 formed a clear rim around the nuclear periphery and distinctive regions of high signal intensity were identified in both 8–16 cell and morula stage embryos (Figure 9a,b). These regions in some areas overlapped with DAPI (the fluorochrome used for DNA labelling: -4,6- diamidino-2-phenylindole). It was observed that DAPI preferably binds to the AT-rich regions which are associated with heterochromatin [47]. Following that labelling property we have used an antibody directed against histone H3 lysine 9 tri-methylation (H3K9me3), which is highly correlated with predominantly transcriptionally silent constitutive heterochromatin regions. The results of double immunofluorescent staining revealed CDX2 and H3K9me3 colocalisation in the interphase nuclei of some cells. Most interestingly in double labelled 8-cell bovine embryos (4 out of 20) we have observed CDX2 localisation at specific regions of the metaphase chromosomes which colocalised with the H3K9me3 (Figure 9a). Also in 4 out of 9 metaphase plates that were altogether detected in 11 morula stage embryos, the specific chromosome coating was also visible (Figure 9b). On close examination we were also able to detect a CDX2 positive signal in a region occupied by a metaphase plate in a single TE cell of late blastocyst (Figure 9c). At this point we would like to stress that data interpretation must be done with much caution and that the possible CDX2 coating of metaphase chromosomes is presented as an observation.

## Discussion

The existing evidence unequivocally indicates species specific differences in cell lineage allocation, as was highlighted by Janet Rossant: “A mouse is not a cow” [48]. However, in light of growing understanding of the signalling processes that control pre-implantation development, we believe that it is more important to ask whether we can identify the same/similar molecular mechanisms that govern cell fate decisions in mouse and in early bovine embryos. To address this question one has to be aware of the species specific differences in early development. The period of mouse preimplantation development is very short and takes 3.5 to 4.0 days. Bovine (and porcine) pre-attachment development is extended in time, as between the blastocyst formation and implantation there is about a 10-day time window during which the embryo undergoes further differentiation. Thus, it may be expected that the events leading to cellular specification and lineage formation in cattle may not be as rapid as in mice. Until now, with some exceptions the authors have concentrated mainly on one developmental stage - the blastocyst at 8/9dpi. Three publications compared transcript abundance between bovine ICM and TE [49-51] however, the approach was mainly qualitative and did not include crucial developmental stages prior to blastocyst formation. Moreover, the existing data is often inconclusive as some authors showed no difference in OCT4 and NANOG protein distribution in bovine ICM and TE [52,53], whereas other [54,55] showed partial OCT4 and NANOG expression in both embryonic lineages. On a contrary Kuijk et al. [56,57] localised NANOG solely in the ICM of bovine blastocysts. Therefore, the aim of our study was to resolve the doubts arising from the analysis of already published material on bovine lineage specification and to identify the time point at which bovine ICM/TE lineage segregation begins.

The first developmental stage investigated was the 5-cell stage which precedes the critical time in bovine development – the transition from maternal to embryonic control of development (MET) [58]. In the mouse MET is initiated at the 2-cell stage. In cattle this process is gradual and may be divided into three stages: (1) early gene activation in zygotes and 2-cell embryos [59]; (2) mid genome activation at 2–5 cell stage accompanied by *de novo* RNA synthesis [60-64] and (3) a major burst of gene activation at the 8–16 cell stage. At the 8–16 cell stage we have detected transcripts for the three canonical lineage markers: *OCT4*, *NANOG* and *CDX2*. The corresponding protein products were noted only for OCT4 and CDX2. Studies on *in vitro* derived bovine embryos revealed a sudden drop in the mRNA level around the 4-cell stage, reaching a minimal value at 8–16 cells [65]. The results obtained here indicate that between the time of EGA and morula formation *NANOG* appear *de novo* as a product of the embryonic genome, as NANOG protein was detected from the morula stage onwards. In support of this assumption *NANOG* transcripts and protein were not detected in bovine germinal vesicle (GV) and *in vitro* matured oocytes [55]. Porcine embryos, which share similar morphological stages of preimplantation development with bovine embryos, display similar mRNA expression patterns for *OCT4* and *CDX2* genes. After EGA, a sudden increase in the mRNA abundance was noted for bovine *OCT4* and *NANOG* and for porcine *OCT4* and *CDX2*[56,66]. *OCT4* transcript expression appears highly conserved between mouse and bovine blastocysts. In both species *OCT4* transcription begins one to two cell cycles after zygotic genome activation and is followed by a sharp increase in the mRNA abundance subsequent to compaction. *OCT4* mRNA was ubiquitously present in all cells of morula stage, however 7dpi (early bovine blastocyst stage) it become restricted to the ICM as indicated by *in situ* hybridisation [49].

Our studies indicate that early in development (5-cell stage) CDX2 and OCT4 proteins are both located in the cytoplasm of bovine blastomeres, however with slightly different distribution patterns. CDX2 was evenly distributed within the cytoplasm and absent in the nuclei. This is different from the expression pattern described in the mouse, where Cdx2 expression becomes detectable from 8–16 cell stage onwards with only nuclear localisation [15]. It indicates that CDX2 expression in cattle precedes the EGA and the stage at which a clear distinction between the inner and the outer cells becomes evident. OCT4 localised at the apical surface of the blastomeres. Asymmetric distribution of *CDX2* transcripts has been shown in the cytoplasm of early mouse embryos (4–8 cell) [66]. Cytoplasmic localisation implies synthesis and accumulation of proteins, thus prior to the EGA bovine embryos may be actively synthesising transcription factors required for lineage specification, what coincides with the gradual process of bovine genome activation. As indicated by our studies at the time of bovine genome activation CDX2 and OCT4 specifically allocate in the nuclei. Cell fate decisions are governed by active interactions between all of the core pluripotency factors, as cells co-express them until a certain threshold is achieved. In the mouse until the late blastocyst stage (3.5/4.0-dpi) CDX2, NANOG and OCT4 proteins exhibit salt and pepper distribution patterns ([41], rev [67]). In the bovine embryo this distribution has been observed, as early blastocysts (7dpi) contained on average 39.5% double positive cells (NANOG and GATA6) [57]. At 8dpi only 6.7% of all NANOG positive cells within the ICM were also positive for GATA6 [57].

As development progresses bovine ICM/TE lineage specification becomes more evident. We have observed that in the early blastocyst (7dpi) at the time of cavitation and spatial separation of the two lineages OCT4 and NANOG occupy the nuclei of the ICM cells. In the neighbouring TE cells however, the proteins have colocalised in the cytoplasm and were absent from the nucleus. This may be the first indication at how functional differences correlate with the cell’s position. In order to be active as transcription factors OCT4 and NANOG need to enter the nucleus, therefore at this stage the proteins may be subjected to an active transportation. OCT4 “rings” observed at the nuclear periphery of the ICM cells may be reflecting this activity. At mid blastocyst stage (8dpi) NANOG and OCT4 colocalised, which is in agreement with studies indicating mutual functional stimulation between these factors [68-71]. At the same developmental stage CDX2 segregation to the TE also became apparent, however some ICM cells still expressed both CDX2 and OCT4. Similarly as has been described in early mouse blastocysts [20], a residual CDX2 signal was detected in the ICM of bovine blastocysts, but in general the ICM was free of CDX2 immunostaining. In late blastocysts we observed OCT4 labelled cells only within the ICM. This has also been observed by Berg et al. [32], who noted measureable amounts of OCT4 in the TE of early bovine blastocysts (7dpi) and none by day 11. The authors indicated that in cattle CDX2 does not repress OCT4 expression, since bovine *OCT4* locus does not contain the *cis*-acting regulatory region necessary for extinguishing transcription in the TE. Nevertheless CDX2 is required for TE maintenance at later developmental stages. This is also true for other species: humans, primates, pigs, horses and rabbits [56,72-75]. Finally, in blastocysts 9dpi we have observed further cellular specification as we have noted cells expressing only NANOG and cells expressing both NANOG and OCT4. Our comparison of relative transcript abundance between bovine ICM and TE corresponds to the protein distribution patterns described here. At the mRNA level we have observed a lineage specific upregulation of *OCT4*, *NANOG* and *FN1* in the *ICM* and *KRT*-*18* in the TE. Notably, the majority of the ICM samples (9/12) lacked the specific transcript for *CDX2*. These results present a similar *CDX2* mRNA expression pattern as has been described for mouse, human and also recently primate embryos [73]. Consequently, within the set of the analysed TE samples 6 out of 12 did not express *NANOG*. Such segregation of key factors responsible for epiblast development may be a prerequisite to further differentiation processes that take place in the bovine embryo between the HBl stage and implantation (around 20dpi). In the mouse, the epiblast formation is defined by two segregation events that depend on the consecutive expression of *Oct4* and *Nanog*. It is therefore plausible that our observations capture the time point at which these processes are initiated in bovine development and indicate that at the molecular level the machinery of cell lineage differentiation is active.

Interestingly factors considered as potential bovine blastocyst formation markers FN-1 and KRT-18 [76,77] also showed lineage specific differences. FN-1 is a glycoprotein component of the extracellular matrix involved in various processes related to early development such as fertilisation, gastrulation and implantation. We have noted *FN*-*1* and *KRT*-*18* transcripts already at the morula stage. FN-1 may be an important factor in the process of compaction and blastocyst formation, as its major biological functions are cell adhesion, cytoskeleton organisation and cell migration. Interactions between all of these processes are essential for early development and cell lineage segregation. Differentiative cell divisions at the morula stage create spatial grounds for the allocation of smaller inner cells to the ICM and larger polarised outer cells to the TE. KRT-18, a cytoskeletal protein, may be seen as a TE specific factor, since targeted deletion of KRT-18 in mouse embryos resulted in trophoblast fragility and early embryonic lethality [78]. We have detected a significant upregulation of *KRT*-*18* gene in bovine TE as compared to the ICM, what makes it an interesting candidate for further investigations of bovine TE lineage formation. Other factors *KLF4*, *c*-*MYC*, *REX1* and *GATA6* did not exhibit lineage specific differences at the mRNA level. Studies showed that *GATA6* mRNA was detected in both PrE and TE outgrowths of bovine blastocysts 8dpi [79]. Furthermore *KLF4* and *c*-*MYC* may not be essential to maintain bovine ICM, since it was indicated that the expression of these two factors alongside OCT4 and SOX2, was not sufficient to induce pluripotency in bovine adult fibroblasts [80]. This may be explained by species specific differences between mouse and cattle as unlike in the mouse, bovine TE is formed from the cells surrounding the blastocyst cavity, not from the ones which cover the ICM [50]. Corresponding to our results Rex1 mRNA was detected in mouse ICM and in polar trophoblast of 4.5dpi blastocyst. Later in mouse development Rex1 was found in trophoblast-derived tissues (ectoplacental cone and extraembryonic ectoderm) of the egg cylinder [81]. Thus Rex1 is regarded as the main factor enabling mouse ESC and epiblast stem cells (EpiSC) to be distinguished.

On a close examination of 8-cell, morula and blastocyst stage embryos we have made a very interesting observation, as in a small proportion of embryos (about 2%) a positive signal for the CDX2 overlapped with the mitotic chromosomes. Moreover, the areas of higher CDX2 intensity in some regions colocalised with DNA labelling fluorochrome (DAPI), which to a certain degree may indicate the differences in chromatin compaction. Because DAPI preferably binds to the AT-rich heterochromatin regions [47] we have used an antibody directed against histone H3 lysine 9 tri-methylation (H3K9me3), which is believed to be associated with the formation of polyploid trophoblast giant cells from diploid trophoblast precursors [82] and together with H3K27 methylation in lysine is correlated with transcriptional repression [83]. In a study of the dynamics of constitutive heterochromatin in bovine nuclear transfer and *in vitro* produced embryos, H3K9me3 colocalised with heterochromatin protein CBX1 (HP1) in 95% of the nuclei and the signal was displayed in patches [84]. This expression pattern was similar to our observations. Double immunofluorescent labelling of 8-cell bovine embryos for CDX2 and H3K9me3 revealed a possible colocalisation of these factors in the interphase nuclei and at the specific regions on the metaphase chromosomes. CDX2 and H3K9me3 colocalisation concurs with the above suggestions, especially since chromatin immunoprecipitation experiments have detected H3K9me3 at numerous promoters in trophoblast tissues. Increased H3K9me3 levels were detected at *Cdx2*, *Eomes* and *Esrrb* promoter regions of ectoplacental cones as compared to extra embryonic endoderm. Thus H3K9me3 may be regarded as a repressive histone marker in early trophoblast development. At this stage data interpretation must be done with much caution, however the phenomenon of mitotic retention of cell fate determining transcription factors, as an epigenetic mechanism regulating gene expression has already been described [85]. At the time of EGA major transcriptional activation takes place. This process directly links chromatin changes, protein allocation and nuclear reorganisation. Mitotic remodelling of cellular and nuclear architecture includes dynamic redistribution of transcription factors (OCT4, ETS1, B-Myb, SP) and degradation of key regulatory proteins (such as cyclins) [86]. After mitosis, the structural and functional identity of cell’s regulatory machinery must be re-established. Enzymatically and chemically sensitive sites on mitotic chromosomes mark active genes. It is therefore plausible that some regulatory complexes remain bound to the condensed chromatin for rapid reactivation after cell division. For example a hematopoietic cell fate determining factor Runx2 remains associated with chromosomes during mitosis through sequence specific chromosome binding [85]. When transcription terminates at the time of chromosome condensation, Runx2 selectively occupies target gene promoters. This allows speculation on possible parallels with the observed CDX2 localisation pattern. There is an intriguing possibility that CDX2 may play a role in the epigenetic regulation of embryonic cell fate through the process of mitotic retention. This is a new finding that is currently a subject of our detailed investigations.

## Conclusions

We have observed that the protein distribution patterns in bovine blastocysts 8-9dpi mostly resemble the mouse model. The differences were noted at earlier developmental stages. In 5 cell embryos CDX2 and OCT4 proteins located in the cytoplasm of bovine blastomeres, but presented slightly different distribution patterns. CDX2 was evenly distributed within the cytoplasm and absent in the nuclei. OCT4 localised at the apical surface of the blastomeres. We have noted changes in sub-cellular and sub-nuclear distribution of pluripotency related factors during the subsequent stages of development. From 8–16 cell stage both proteins displayed a nuclear localisation, but from the morula stage the specific lineage segregation became visible as OCT4 became ICM specific and CDX2 located in the TE layer. NANOG was initially detected in the cytoplasm of certain cells of the morula stage embryos and became ICM specific at 8dpi. Finally it may be speculated that CDX2 possibly occupies transcriptionally silent regions of the early bovine nuclei as it was found to colocalise with about 2% of the observed metaphase plates.

## Competing interests

The authors declare no competing interests.

## Authors’ contributions

ZEM is the grant holder, designed and performed the research, analysed the data and wrote the manuscript; JS performed immunofluorescent staining experiments and was involved in confocal analyses; KH and EW were involved in IVP experiments; PP helped with confocal imaging; NR and DL contributed to data analysis; BP was involved in research development and participated in manuscript preparation. All authors read and approved the final manuscript.

## Supplementary Material

Additional file 1: Figure S1 Control staining of bovine blastocysts subjected to the same immunolabelling protocol as the experimental group, with the exception of primary antibodies. Scale bar = 50 μm. Click here for file
