# Supplementary figures and images for: Changes in sub-cellular localisation of trophoblast and inner cell mass specific transcription factors during bovine preimplantation development
Source: BMC Dev Biol. 2013 Aug 13;13:32. doi: 10.1186/1471-213X-13-32 (PMC3751447; doi:10.1186/1471-213X-13-32)

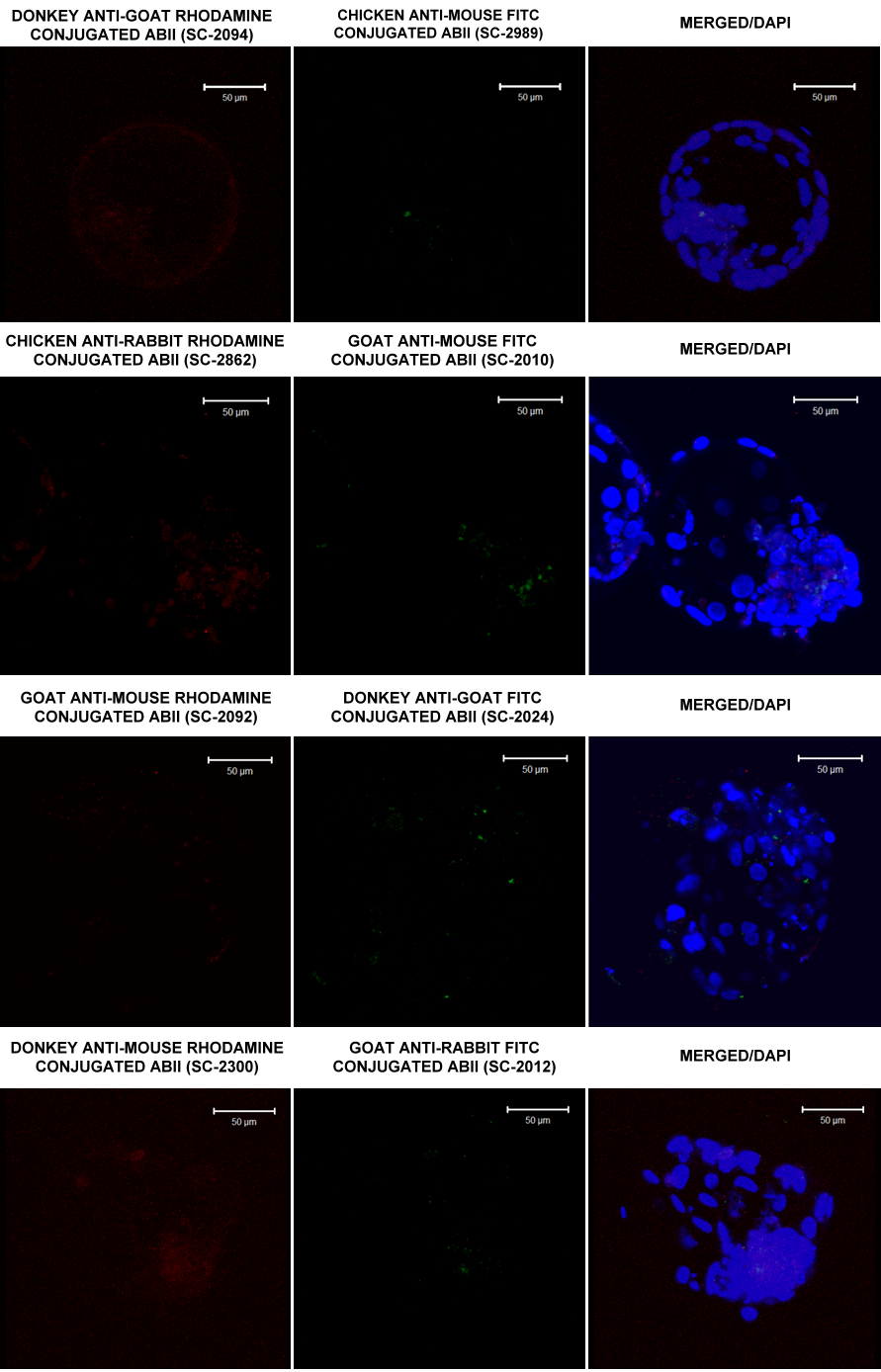

Supplement: Additional file 1: Figure S1 — Control staining of bovine blastocysts subjected to the same immunolabelling protocol as the experimental group, with the exception of primary antibodies. Scale bar = 50 μm. [file 1471-213X-13-32-S1.tiff]
